# Platelet CLEC-2 protects against lung injury via effects of its ligand podoplanin on inflammatory alveolar macrophages in the mouse

**DOI:** 10.1152/ajplung.00023.2017

**Published:** 2017-08-24

**Authors:** Siân Lax, Julie Rayes, Surasak Wichaiyo, Elizabeth J. Haining, Kate Lowe, Beata Grygielska, Ryan Laloo, Per Flodby, Zea Borok, Edward D. Crandall, David R. Thickett, Steve P. Watson

**Affiliations:** ^1^Institute of Cardiovascular Science, College of Medical and Dental Sciences, University of Birmingham, Edgbaston, Birmingham, United Kingdom;; ^2^Will Rogers Institute Pulmonary Research Center and Division of Pulmonary, Critical Care and Sleep Medicine, Department of Medicine, Keck School of Medicine, University of Southern California, Los Angeles, California; and; ^3^Institute of Inflammation and Ageing, University of Birmingham Research Labs, QE Hospital, Birmingham, United Kingdom

**Keywords:** acute respiratory distress syndrome, platelets, alveolar macrophages, mouse models

## Abstract

There is no therapeutic intervention proven to prevent acute respiratory distress syndrome (ARDS). Novel mechanistic insights into the pathophysiology of ARDS are therefore required. Platelets are implicated in regulating many of the pathogenic processes that occur during ARDS; however, the mechanisms remain elusive. The platelet receptor CLEC-2 has been shown to regulate vascular integrity at sites of acute inflammation. Therefore the purpose of this study was to establish the role of CLEC-2 and its ligand podoplanin in a mouse model of ARDS. Platelet-specific CLEC-2-deficient, as well as alveolar epithelial type I cell (AECI)-specific or hematopoietic-specific podoplanin deficient, mice were established using *cre-loxP* strategies. Combining these with intratracheal (IT) instillations of lipopolysaccharide (LPS), we demonstrate that arterial oxygen saturation decline in response to IT-LPS in platelet-specific CLEC-2-deficient mice is significantly augmented. An increase in bronchoalveolar lavage (BAL) neutrophils and protein was also observed 48 h post-IT-LPS, with significant increases in pro-inflammatory chemokines detected in BAL of platelet-specific CLEC-2-deficient animals. Deletion of podoplanin from hematopoietic cells but not AECIs also reduces lung function and increases pro-inflammatory chemokine expression following IT-LPS. Furthermore, we demonstrate that following IT-LPS, platelets are present in BAL in aggregates with neutrophils, which allows for CLEC-2 interaction with podoplanin expressed on BAL inflammatory alveolar macrophages. Taken together, these data suggest that the platelet CLEC-2-podoplanin signaling axis regulates the severity of lung inflammation in mice and is a possible novel target for therapeutic intervention in patients at risk of developing ARDS.

## INTRODUCTION

Acute respiratory distress syndrome (ARDS) is a devastating clinical syndrome of acute respiratory failure in the critically ill. It is the final common pathway of response to a variety of direct pulmonary insults such as bacterial/viral pneumonia and gastric aspiration, or indirect insults such as abdominal sepsis or battlefield trauma (10, 35, 45). Under the Berlin definition for ARDS, onset of diagnosis must be within 7 days, with bilateral opacities present on chest X-ray and severity defined as “mild” ($Pa_{O}{}_{{}_{2}}$/$FI_{O}{}_{{}_{2}}$= 200–300), “moderate” ($Pa_{O}{}_{{}_{2}}$/$FI_{O_{2}}$= 100–200), or “severe” ($Pa_{O}{}_{{}_{2}}$/$FI_{O_{2}}$< 100) (10, 53). The incidence of ARDS has been reported to range from 7.2 to 78.9 cases per 100,000 worldwide, with mortality estimated at ~40% and pulmonary impairment persisting in up to 50% of survivors, leading to enormous social and fiscal cost (45, 49, 55). There are no current readily available tests that can clearly identify those who are at high risk of ARDS, and no therapeutic interventions proven to prevent its occurrence. Clearly other mechanistic insights into the pathophysiology of ARDS are needed to identify new pathways for therapeutic manipulation.

Traditionally thought of as key regulators of physiological and pathogenic hemostasis, platelets are now recognized as essential mediators of both innate and adaptive immunity (22–24, 52). Platelets are implicated in regulating many of the pathogenic processes that occur during ARDS, including neutrophil recruitment, macrophage-dependent inflammation, and alveolar-capillary permeability through complex mechanisms (37). Murine models suggest that thrombocytopenia is protective during sterile lipopolysaccharide (LPS)-induced lung inflammation, primarily by reducing neutrophil recruitment (16, 30). However, this would presumably be detrimental if the bactericidal activities of neutrophils are required. Indeed, thrombocytopenia in intensive care unit patients is associated with an increased risk of ARDS (56). In addition, severe thrombocytopenia (<5 × 10^9^ platelets/l) in a mouse model of pneumonia-induced sepsis enhanced pro-inflammatory cytokine release and significantly impaired survival (5).

Platelets are required to maintain endothelial barrier function under both homeostatic and inflammatory conditions. Looney et al. (30) originally demonstrated a pathogenic role for platelets in an anti-MHC I and LPS-induced murine transfusion-related acute lung injury (TRALI) model. A more recent publication, however, questioned the role of platelets in the initiation and development stages of the TRALI model, with only red blood cell accumulation/alveolar hemorrhage argued to be platelet-dependent because of their role in regulating vascular integrity (19). Maintenance of vascular integrity is primarily driven via activation of and signaling through the immunoreceptor tyrosine-based activation motif (ITAM)-containing receptors (29). Murine platelets express two ITAMs: GPVI and the hemITAM C-type lectin-like 2 (CLEC-2), both of which are critical for securing vascular integrity within the lungs during inflammation (2). Whereas GPVI is restricted to platelets, CLEC-2 expression has been shown to be present at a low level on a small subset of inflammatory cells (33, 39). To date, the only known endogenous ligand for CLEC-2 is podoplanin (also known as gp38 or T1α), a cell surface protein originally described on kidney podocytes and alveolar epithelial type I cells (AECIs) (3, 51) that is also expressed on lymphatic endothelial cells (43), inflammatory macrophages (26), CD4 T cell subsets (48), and stromal cells (11). Mice deficient in either CLEC-2 or podoplanin exhibit separation defects of the blood and lymphatic systems, and present with significantly altered lung function that may contribute to their perinatal lethality (12, 50). Furthermore, platelet activation via CLEC-2 is independent of major hemostatic pathways and thus is a candidate for the development of novel therapies in lung injury (40).

On the basis of the expression of podoplanin in the lung, the unusual role of CLEC-2 in platelet activation and the phenotype of CLEC-2 or podoplanin-deficient mice, we hypothesized that the CLEC-2-podoplanin axis may play an as yet unrealized role in ARDS. In this study we investigate the in vivo consequences on acute lung injury of deleting CLEC-2 or podoplanin by using a mouse model of ARDS. We show that LPS-induced lung inflammation leads to recruitment of platelets predominantly bound to neutrophils into alveoli. Furthermore, CLEC-2 expressed on platelets is required to limit lung function decline as assessed by arterial oxygen saturation, confirmed by increases in neutrophilia and BAL protein observed in CLEC-2 deficient animals. We further demonstrate that this action is mediated by interaction of CLEC-2 with its ligand podoplanin expressed on a macrophage population (CD11b^+^CD11c^+^F4/80^+^) only present in alveoli during inflammatory conditions. Taken together, these data suggest that the platelet CLEC-2-podoplanin signaling axis is protective during a model of mouse lung injury, and is therefore a possible novel target for future therapeutic intervention in patients at risk of developing ARDS.

## MATERIALS AND METHODS

### 

#### Mice.

All mice were maintained in individually ventilated cages under a 12-h:12-h light/dark cycle at a constant temperature of 20°C with food and water given ad libitum at the Biomedical Services Unit, Birmingham University, UK. All experiments were performed in accordance with UK laws [Animal (Scientific Procedures) Act 1986] with approval of local ethics committee and UK Home Office approval under PPL 40/3741. *Clec2*^fl/fl^ mice with either PF4cre, Rosa26-ER^T2^cre (ER^T2^cre), or CD11c-cre along with *Pdpn*^fl/fl^ VAV1cre^+^ have been described previously (1, 12, 20, 33). Animals were fed with FormulaLab Diet 5008 (Laboratory-Diet, St. Louis, MO). When required, 4- to 6-wk-old *Clec2*^fl/fl^ER^T2^cre and their *Clec2*^fl/fl^ control littermates were fed for 2 wk with tamoxifen-supplemented diet TAM 400 (Envigo, UK), and then returned to the FormulaLab Diet 5008 chow. Mice expressing Aquaporin 5 cre (Aqp5cre) (13) were crossed with mice expressing the podoplanin floxed allele to generate the *Pdpn*^fl/fl^Aqp5cre^+^ strain. Wild-type C57Bl/6 mice were purchased from Harlan Laboratories (Oxford, UK). Male mice, aged 9–16 wk, were used.

#### Intratracheal LPS model.

Instillations of 40 μg LPS (*Escherichia coli* O111:B4, InvivoGen, France) in a 50-μl bolus of phosphate-buffered saline (PBS) or PBS alone were administered to each mouse. Mice were euthnaized at 48 h post-LPS instillation (peak of cellular recruitment), or following resolution of the inflammatory response, 9 days post-LPS instillation (28). Infrared pulse oximetry and pulse distention were assessed by MouseOx Plus (Starr Life Sciences) and bronchoalveolar lavage (BAL) collected as previously described (28). The epithelial damage marker receptor for advanced glycation end products (RAGE), as well as cytokine and chemokine levels were analyzed using Fluorokine MAP Multiplex (R&D Systems, UK). Whole blood was collected into ethylenediaminetetraacetic acid (EDTA), analyzed on an ABX Pentra 60 (Horbia, UK), and plasma was isolated by centrifugation at 2,000 *g* for 30 min.

#### Lung water and protein permeability.

Protein permeability was calculated by measuring the concentration of total protein in BAL samples per milliliter of fluid recovered. To calculate wet wt-to-dry wt ratio, mice were culled by cervical dislocation and lungs removed en bloc. Heart, thymus, and connective tissue were removed, and “wet” lungs weighed. Lungs were dried in a 55°C oven for 48 h. The resulting “dry” lungs were weighed, and the ratio between the two values determined.

#### Platelet depletion.

Intraperitoneal injections of 1.5 μg/g anti-mouse GP1bα antibody (EMFRET analytics, Germany; R300) were given 18 h before IT-LPS. Complete and sustained platelet depletion was confirmed in whole blood immediately before IT-LPS; 25 ± 10 × 10^3^/mm^3^ compared with 959 ± 34 × 10^3^/mm^3^ isotype controls (~97%).

#### Platelet transfusion.

Blood was collected from the inferior vena cava of wild-type C57Bl/6 mice under terminal anesthesia, and platelets were prepared as previously described (46). Blood was pooled from a number of donor mice, transfused into recipient *Clec2*^fl/fl^ or *Clec2*^fl/fl^ER^T2^cre^+^ mice (2 × 10^8^ in 200 μl of buffer via the tail vein). Immediately after intravenous injections, recipient mice were subjected to IT-LPS. The platelets to be transfused were confirmed to be inactive, with the potential to become fully activated by analyzing P-selectin and fibrinogen binding via flow cytometry following incubation with or without 0.5 U/ml thrombin (Sigma-Aldrich, UK) for 15 min at room temperature.

#### Lung digestion and flow cytometry.

Lungs were perfused with 10 ml of 200 nM EDTA/PBS until white. BAL was extracted and the left lobe digested using Liberase TL (0.4 Wunsch units/ml; Roche) with DNase (50 μg/ml; Roche) and incubated at 37°C in a rotating shaker (200 rpm) for 40 min. Single-cell suspensions were ensured by passing through a 25-G needle four times and washing the cells through a 70-μm filter. Pelleted cells from BAL were first enumerated and then assessed by flow cytometry alongside lung tissue cells by using fluorophore-conjugated antibodies (eBioscience). Only SyTOX (Invitrogen) negative cells (live cells) from digested tissue were analyzed. BAL neutrophil and red blood cell numbers are presented per milliliter of BAL. Cell populations from digested tissue were stained as previously described (18) or as stated in the text.

#### Lung histology and podoplanin expression.

Lungs were inflated postmortem with 1.5 ml of a solution containing optimum cutting temperature compound (Tissue-Tek, The Netherlands) in PBS (1:2) introduced via the trachea by using a 19-G needle. The trachea was then sutured and lungs removed en bloc, frozen over dry ice, and stored at −80°C. Frozen sections of 20 μm were processed for hematoxylin and eosin or podoplanin staining (clone 8.1.1., eBioscience). Syrian hamster IgG controls were used to confirm specific staining (data not shown). Images were analyzed using a Zeiss Axio Scan.Z1 microscope and ZEN software.

#### Statistical analysis.

All parameters were analyzed using Prism 6 (GraphPad Software). Significance was assessed by ANOVA with relevant posttest analysis as indicated in the text. Data are presented as Box-Whisker plots with mean displayed and the range of minimum to maximum data points.

## RESULTS

### 

#### Intratracheal LPS induces recruitment of platelet-neutrophil aggregates into lung alveoli.

**Fig. 1.**
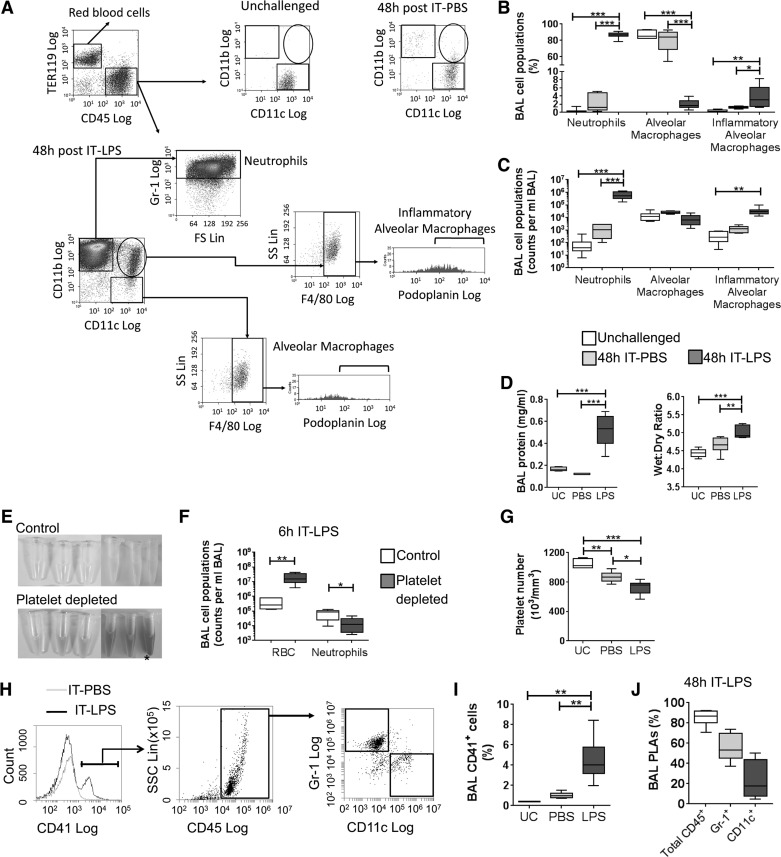
Platelets are present in bronchoalveolar lavage (BAL) during a mouse model of acute respiratory distress syndrome (ARDS). Unchallenged BAL and BAL isolated 48 h after intratracheal phosphate-buffered saline (IT-PBS) or lipopolysaccharide (LPS) were analyzed from wild-type (WT) mice by flow cytometry. *A*: representative histograms for the identification of leucocyte (CD45^+^) cell populations in mouse BAL before and 48 h after IT-PBS or LPS. [From Duan et al. (9).] Podoplanin expression was analyzed by setting the gate at ≤1% by using a Syrian hamster IgG isotype. FS, forward scatter; SS, side scatter. The major leucocyte populations observed in BAL displayed as percentage of total CD45^+^ BAL cells (*B*) and absolute cell counts (*C*); *n* = 8. *D*: alveolar-capillary damage assessed in unchallenged mice and 48 h after IT-PBS or LPS; *n* = 6–8. *E*: BAL from WT mice pretreated with isotype (control) or anti-GP1bα antibody (platelet depleted) mice 6 h after IT-LPS; *excluded animal. *F*: the numbers of red blood cells (RBCs) and neutrophils in BAL of WT mice pretreated with isotype (control) or anti-GP1bα antibody (platelet depleted) mice 6 h after IT-LPS. Isotype control *n* = 6, anti-GP1bα *n* = 5; Student’s *t-*test. *G*: systemic platelet counts from WT mice where analyzed in whole blood on an ABX Pentra 60 (Horbia) in unchallenged mice or 48 h after IT-PBS or LPS; *n* = 8. *H*: representative histograms for the identification of CD41^+^ platelets in BAL and characterization of platelet-leucocyte aggregates (PLA) following IT-LPS. *I*: CD41^+^ platelets observed in BAL in WT mice 48 h following IT-LPS. UC, unchallenged. *J*: identification of CD41^+^CD45^+^ PLAs in BAL 48 h following IT-LPS; *n* = 8. One-way ANOVA performed with Tukey’s multiple comparison posttests; *P < 0.05, **P < 0.01, and ***P < 0.001.

When assessed by flow cytometry, BAL from unchallenged mice contains predominantly alveolar macrophages (CD11b^−^CD11c^+^F4/80^+^). As expected, following IT-LPS the percentage of neutrophils (CD11b^+^CD11c^−^Gr1^+^) increases dramatically in BAL, along with an inflammatory macrophage population (CD11b^+^CD11c^+^F4/80^+^) (Fig. 1, *A* and *B*). As a consequence resident alveolar macrophages are reduced in percentage. When enumerated, the numbers of tissue resident alveolar macrophages remain constant, with increases in neutrophils and inflammatory alveolar macrophages observed (Fig. 1*C*). Total BAL protein and lung wet wt-to-dry wt ratio confirms that IT-LPS induces significant lung damage compared with PBS-treated or unchallenged controls (Fig. 1*D*). No significant differences are observed between unchallenged and IT-PBS treated mice.

The critical role of platelets during LPS-induced lung injury has previously been demonstrated in platelet-depleted mice by using administration of a rat anti-mouse GP1bα monoclonal antibody (7, 15). In our model we confirm these data and observe that pretreatment of wild-type mice with anti-GP1bα antibody significantly increases bleeding into the alveoli, as assessed by red blood cell (RBC) accumulation in BAL compared with isotype-treated controls following IT-LPS (53.9 fold; Fig. 1, *E* and *F*). Concomitantly, the number of neutrophils observed in BAL is significantly reduced in platelet-depleted animals following IT-LPS (4.5 fold; Fig. 1*G*). As a consequence, anti-GP1bα-treated mice present with an irregular breathing pattern and reduced activity 6 h after LPS administration compared with isotype-treated controls. One anti-GP1bα antibody-treated animal had to be culled 4 h post-IT-LPS because of welfare concerns (* in Fig. 1*E*). This animal was excluded from analysis.

In wild-type mice with normal platelet counts, IT-PBS induces a decrease in platelet count by 17% compared with unchallenged controls. Platelet count drops 13% further following IT-LPS (Fig. 1*G*), coinciding with the emergence of a CD41^+^ platelet population within the BAL, not seen in control or IT-PBS-treated BAL (Fig. 1, *H* and *I*). Flow cytometry reveals that the majority of these platelets are within platelet-leucocyte aggregates (PLAs; 80.8 ± 5.3%), primarily bound to Gr-1^+^ neutrophils (58.3 ± 5.8%) (Fig. 1*J*). These data demonstrate the location of a population of platelets, which express CLEC-2, within the alveoli during IT-LPS-induced inflammation.

#### Platelet-expressed CLEC-2 protects against LPS-induced lung injury.

**Fig. 2.**
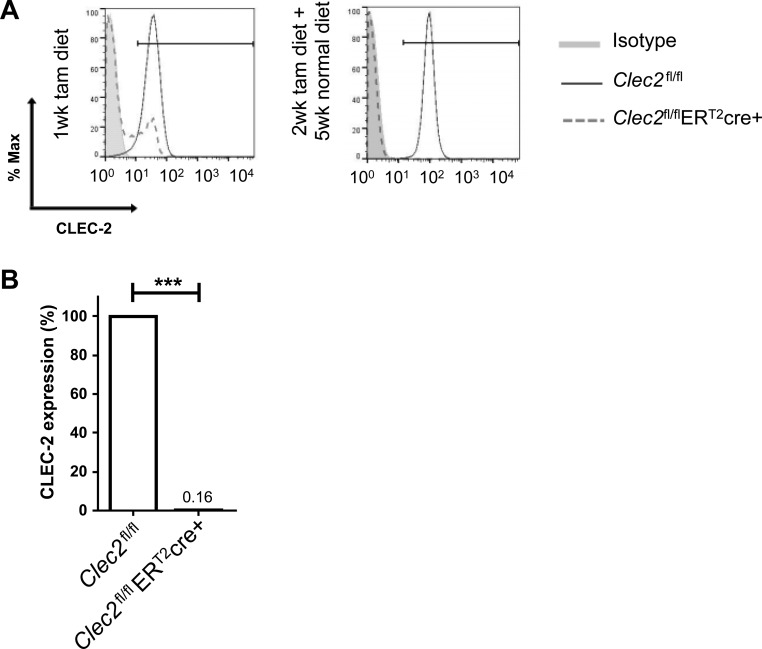
Two-week tamoxifen-induced deletion of C-type lectin-like 2 (CLEC-2) on platelets. *A*: representative histograms using 17D9-FITC to detect CLEC-2 expression on platelets in whole blood analyzed 1 wk after starting tamoxifen diet (tam) and after 2-wk tamoxifen diet followed by 5 wk of normal diet in *Clec2*^fl/fl^ER^T2^cre mice compared with floxed only controls. An IgG2b-FITC isotype control was also included. *B*: percentage of CLEC-2 expression in CD41 positive platelets was analyzed by flow cytometry. One-way ANOVA with Tukey’s multiple posttests; *n* = 4; ***P < 0.001.

Constitutive deletion of CLEC-2 is perinatally lethal (12). Therefore, we used two *cre-loxP* strategies to specifically delete CLEC-2 from platelets in mice. First a rosa26-driven tamoxifen-inducible (ER^T2^) cre was used. When a tamoxifen diet is given for only 2 wk, recombination in the megakaryocyte lineage allows for newly synthesized “knockout” platelets to be produced because of their relatively short circulation time of 5 days (33) (Fig. 2). This is followed by a 4–5 wk “washout” period of normal diet to ensure the anti-inflammatory effects of tamoxifen do not alter responses to LPS. A PF4cre was used to delete CLEC-2 specifically in the megakaryocyte/platelet lineage (12).

**Fig. 3.**
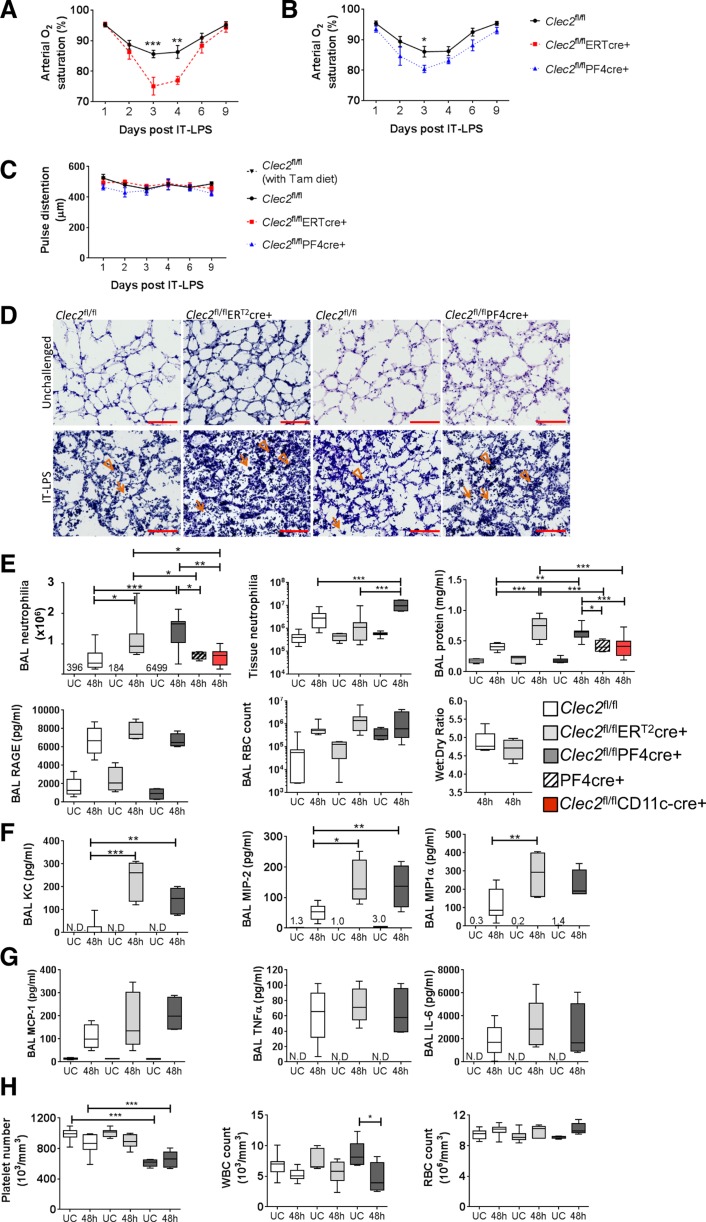
Effect of CLEC-2 deletion in platelets during a mouse model of ARDS. Arterial oxygen saturation analyzed by noninvasive pulse oximetry following IT-LPS in tamoxifen (ER^T2^)cre- or PF4cre-induced CLEC-2-deficient mice compared with cre negative controls (*A* and *B*, respectively). Tamoxifen-treated *Clec2*^fl/fl^ vs. *Clec2*^fl/fl^ER^T2^cre *P* < 0.001; *Clec2*^fl/fl^ vs. *Clec2*^fl/fl^PF4cre; *P* < 0.001. *C*: pulse distention analyzed by MouseOx Plus following IT-LPS in tamoxifen (ER^T2^)- or PF4cre-induced CLEC-2-deficient mice compared with cre negative controls. Tamoxifen-treated *Clec2*^fl/fl^: *n* = 8; *Clec2*^fl/fl^ER^T2^cre: *n* = 7; *Clec2*^fl/fl^: *n* = 8; *Clec2*^fl/fl^PF4cre: *n* = 8; means ± SE. Two-way ANOVA performed with Tukey’s multiple comparisons test. *D*: representative images of lung sections stained for hematoxylin and eosin in unchallenged and 48 h after IT-LPS-treated ER^T2^cre- or PF4cre-induced CLEC-2-deficient mice compared with cre negative controls. Alveolar and interstitial neutrophils are highlighted by arrows and arrowheads, respectively. Scale bar: 100 μm; *n* = 3–5. *E*: neutrophil recruitment and pulmonary endothelial and epithelial damage parameters in unchallenged and IT-LPS-treated ER^T2^cre- or PF4cre-induced CLEC-2-deficient mice compared with cre negative controls; *n* = 7–13. PF4cre only, *n* = 4; *Clec2*^fl/fl^CD11c-cre, *n* = 8. *F *and *G*: bronchoalveolar lavage (BAL) cyto- and chemokine levels in unchallenged and IT-LPS-treated ERT2cre- or PF4cre-induced CLEC-2-deficient mice compared with cre negative controls; *n *= 6. *H*: systemic platelet, white blood cell (WBC), and red blood cell (RBC) counts were analyzed in whole blood on an ABX Pentra 60 (Horbia); *n *= 6–11. One-way ANOVA performed with Tukey’s multiple comparison posttests; *P < 0.05, **P < 0.01, and ***P < 0.001. UC, unchallenged; N.D., not detected; 48 h = 48 h post-IT-LPS.

To investigate the consequence of platelet-CLEC-2 deletion, we first analyzed arterial oxygen saturation ($Sa_{O_{2}}$) via pulse oximetry to assess overall lung function. Instillation of PBS in CLEC-2-deficient animals or littermate controls does not alter $Sa_{O_{2}}$, which is maintained at ~96% (data not shown). After IT-LPS, deletion of CLEC-2 significantly reduces $Sa_{O_{2}}$ in both genetically altered strains compared with floxed controls (*P* < 0.001; Fig. 3, *A* and *B*). There is no significant difference between the CLEC-2-deficient strains (*P* = 0.34) or between floxed-only controls given a normal or tamoxifen diet (*P* = 0.54). In addition, there are no significant differences in pulse distention, indicative of blood flow (41), between these strains (Fig. 3*C*).

Immunohistochemistry demonstrates an increase in accumulation of both alveolar and interstitial neutrophils in CLEC-2-deficient animals 48 h post-IT-LPS compared with littermate controls (Fig. 3*D*, arrowheads and arrows, respectively). To quantify this, we analyzed BAL neutrophil counts. A significant increase in alveolar neutrophilia 48 h post-IT-LPS in both models of CLEC-2-deficient mice was observed compared with littermate controls (2.3–2.9 fold; Fig. 3*E*). In addition, quantification of interstitial neutrophilia suggests that CLEC-2 deletion driven by PF4cre leads to an increase in the number of neutrophils within lung tissue (Fig. 3*E*).

A significant increase in BAL protein is observed in both CLEC-2-deficient strains compared with controls 48 h post-IT-LPS (1.5–1.7 fold; Fig. 3*E*). However, no significant differences are observed in wet wt-to-dry wt ratio, expression of the epithelial cell damage marker, RAGE, or RBC accumulation in BAL (alveolar hemorrhage) 48 h post-IT-LPS in CLEC-2-deficient strains compared with controls (Fig. 3*E*). These data suggest that the increases in BAL protein observed may reflect the increase in protein derived from inflammatory cells rather than an increase in lung permeability in CLEC-2-deficient animals (36).

When using *cre-loxP* technology, nonspecific endonuclease activity of the cre recombinase is always a concern (31). However, we observe no cre-related effects on neutrophil recruitment or BAL protein using PF4cre only expressing mice 48 h post-IT-LPS (Fig. 3*E*, striped plots). This suggests our data are specific to CLEC-2 deletion. Furthermore, as a cell type previously reported to express CLEC-2 and to have a significant role during inflammation, we used the previously characterized *Clec2*^fl/fl^CD11c-cre mice to assess the contribution of CLEC-2 expressed on dendritic cells (1). We again observe no effects on neutrophil recruitment or BAL protein using the dendritic cell-specific CLEC-2-deficient mice (Fig. 3*E*, red plots).

Expression of the murine neutrophil recruitment chemokines, keratinocyte-derived chemokine (KC) and macrophage inflammatory protein 2 (MIP-2), in BAL are significantly increased in CLEC-2-deficient animals (2.6–3.0 and 8.8–14.8 fold, respectively) (Fig. 3*F*). However, no significant difference was observed in expression of the monocyte recruitment chemokine MCP-1 or pro-inflammatory cytokines, tumor necrosis factor (TNF)α, and interleukin (IL)-6 (Fig. 3*G*). Platelet numbers in whole blood from *Clec2*^fl/fl^ER^T2^cre^+^ mice, along with white blood cell (WBC) and RBC counts of all cre^+^ mice, were comparable to floxed-only controls (Fig. 3*H*). *Clec2*^fl/fl^PF4cre^+^ mice have a mild reduction in platelet numbers (~13–23%) due to blood-lymphatic mixing, but this does not affect WBC or RBC, as previously reported (12). Together these data suggest that platelet CLEC-2 is protective during IT-LPS.

**Fig. 4.**
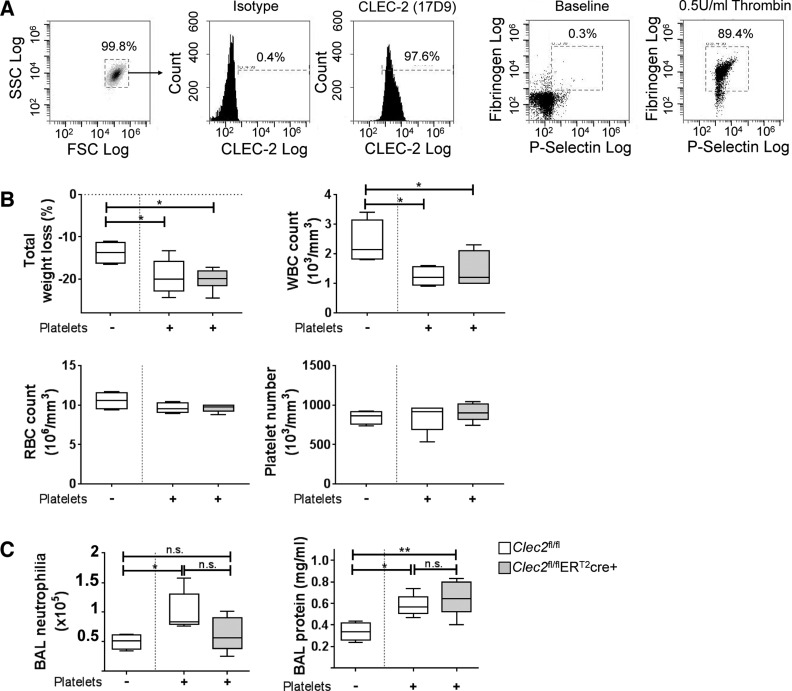
Effect of platelet transfusion during a mouse model of ARDS. *A*: purity and CLEC-2 expression and activity were confirmed in platelets prepared from WT mice. These platelets were given via intravenous injection (2 × 10^8^ per mouse) immediately before LPS instillations into CLEC-2 sufficient (*Clec2*^fl/fl^) and platelet-specific CLEC-2-deficient (*Clec2*^fl/fl^ER^T2^cre^+^) mice. Control and CLEC-2-sufficient mice that were given IT-LPS on the same day but did not receive platelets were also included. *B*: total weight loss along with systemic white blood cell (WBC), red blood cell (RBC), and platelet counts in whole blood were analyzed 48 h post-IT-LPS. *C*: bronchoalveolar (BAL) neutrophilia and total BAL protein were also analyzed 48 h post-IT-LPS. One-way ANOVA performed with Tukey’s multiple comparison posttests; *Clec2*^fl/fl^ (no transfusion) *n* = 4, *Clec2*^fl/fl^^+^ platelets *n* = 5, *Clec2*^fl/fl^ER^T2^cre^+^ platelets *n* = 7; *P < 0.05 and **P < 0.01; n.s. = not significant.

A recent publication utilizes a simple platelet transfusion to confirm the role of platelet CLEC-2 during a mouse model of deep vein thrombosis (DVT) (46). Therefore, using a similar strategy we sought to confirm that the dysregulation of lung inflammation in the CLEC-2-deficient strains is due to the lack of CLEC-2 on platelets. Purity, CLEC-2 expression, and potential activation of the purified wild-type platelets used in these experiments were first confirmed by flow cytometry (Fig. 4*A*). Although simple in concept, platelet transfusion within an inflammatory model such as IT-LPS, however, leads to a complex phenotype. Platelet transfusion immediately before IT-LPS into CLEC-2-deficient or sufficient controls has a negative impact on animal welfare, as evidenced by a significant decrease in total body weight loss and a pronounced systemic leukopenia 48 h post-IT-LPS compared with mice that did not receive a transfusion (Fig. 4*B*). Transfusions did not alter systemic numbers of RBCs or platelets in whole blood 48 h after IT-LPS.

In wild-type *Clec2*^fl/fl^ controls, platelet transfusion significantly increases neutrophil recruitment to the alveoli along with a significant increase in total BAL protein 48 h post-IT-LPS compared with mice that did not receive platelets (Fig. 4*C*). When platelet transfusions were given to platelet-specific CLEC-2-deficient animals (*Clec2*^fl/fl^ER^T2^cre^+^), although a trend for reduced neutrophilia was observed following IT-LPS, their response was not significantly different to *Clec2*^fl/fl^ mice that also received platelets (Fig. 4*C*, gray plots). In addition, no difference in total BAL protein was observed comparing *Clec2*^fl/fl^ and *Clec2*^fl/fl^ER^T2^cre^+^ animals that had all received platelets.

The altered response observed in the control mice that received platelets makes subsequent analysis of CLEC-2-deficient animals problematic. Having observed that without platelet transfusion, CLEC-2-deficient mice have increased BAL neutrophilia and protein, one may assume that this would also be upheld following the addition of a platelet transfusion. The fact that we do not observe any additive effects of lacking platelet CLEC-2 in mice that receive CLEC-2-expressing platelets may suggest that the platelet transfusion has indeed “rescued” the defect in deficient mice. This supports our findings that it is CLEC-2, specifically expressed on platelets, that is required to limit pulmonary inflammation during IT-LPS. However, this explanation may be too simplistic, with the possibility that platelet transfusion alters and/or overcomes any CLEC-2-dependent signaling.

#### Podoplanin expression in Aqp5cre and VAV1cre conditional knockout lungs.

The only known endogenous ligand for CLEC-2 is podoplanin. Like CLEC-2, constitutive deletion of podoplanin is lethal in murine models (50). Therefore we generated two conditional knockout strains by using a floxed podoplanin allele [*Pdpn*^fl/fl^ (32)]. We first used previously characterized Aqp5cre mice (13) to generate a novel AECI-specific podoplanin-deficient strain. In addition, we used the Vav1cre strain to generate hematopoietic-specific podoplanin-deficient mice (20). Both strains are viable, without any overt phenotype (Table 1) and exhibit Mendelian inheritance (Table 2).

**Table 1.** Phenotype of Pdpn^fl/fl^Aqp5cre and Pdpn^fl/fl^VAV1cre mouse strains

Parameter	*Pdpn*^fl/fl^	*Pdpn*^fl/fl^Aqp5cre	*Pdpn*^fl/fl^VAV1cre	*P*
Body weight, g	28.8 ± 1.7	27.8 ± 0.7	27.1 ± 2.8	0.338
Normalized organ weight				
Lungs	11.5 ± 1.0	11.2 ± 0.4	11.5 ± 0.7	0.740
Heart	5.6 ± 0.4	5.5 ± 0.5	5.4 ± 0.4	0.829
Spleen	2.9 ± 0.4	3.0 ± 0.2	2.7 ± 0.2	0.206
Thymus	1.8 ± 0.3	2.0 ± 0.2	1.9 ± 0.5	0.787
Systemic platelet count, 10^3^/mm^3^	858 ± 82	822 ± 87	847 ± 66	0.700
CLEC-2 expression on platelets, %	99.1 ± 0.5	99.0 ± 0.7	98.7 ± 0.8	0.599
*n*	6	6	6	

Values are means ± SD with one-way ANOVA. Male mice 10.5–12 wk old were analyzed and compared with *Pdpn*^fl/fl^ littermate controls. Organ weights were normalized to body weight.

**Table 2.** Genetic inheritance in Pdpn^fl/fl^Aqp5cre and Pdpn^fl/fl^VAV1cre mouse strains

		Genotype					
Breeding Pair	Sex	*Pdpn*	Cre	Total	Expected	Observed	*P*
*Pdpn*^fl/fl^ X *Pdpn*^fl/fl^Aqp5cre				109			
	M	fl/fl	—		27.25	22	0.162
	M	fl/fl	Aqp5		27.25	32	
	F	fl/fl	—		27.25	25	0.579
	F	fl/fl	Aqp5		27.25	29	
*Pdpn*^fl/fl^ X *Pdpn*^fl/fl^VAV1cre				247			
	M	fl/fl	—		61.75	67	0.173
	M	fl/fl	VAV1		61.75	53	
	F	fl/fl	—		61.75	63	0.747
	F	fl/fl	VAV1		61.75	64	

No significant differences in terms of inheritance in either strain or within sexes were detected by χ^2^-test.

**Fig. 5.**
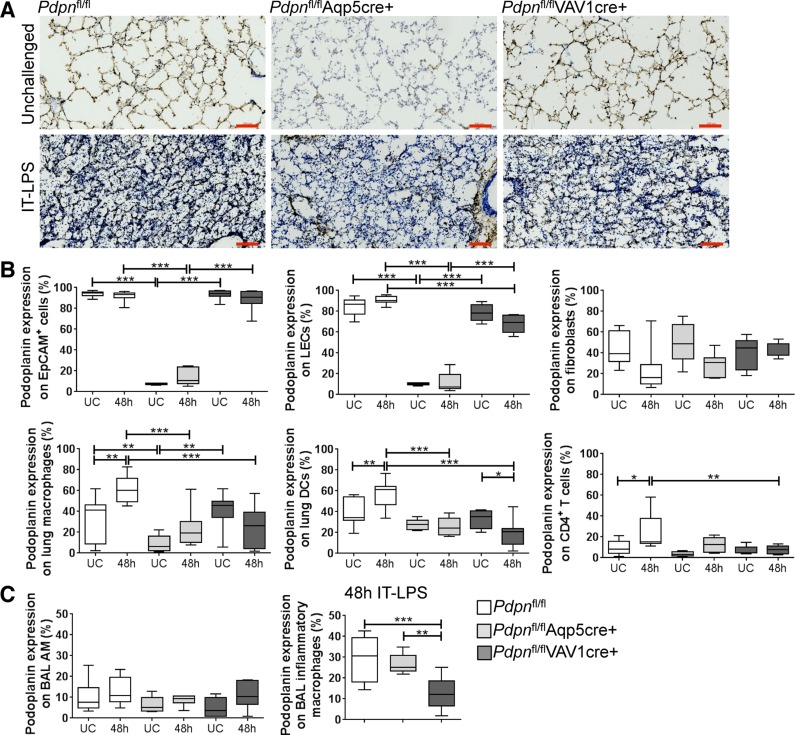
Podoplanin expression in unchallenged and IT-LPS-treated *Pdpn*^fl/fl^ Aqp5cre and VAV1cre mice. *A*: representative images of lung sections stained using 8.1.1 to detect podoplanin (brown) and hematoxylin (blue) in unchallenged and 48 h after IT-LPS-treated Aqp5cre- or VAV1cre-induced podoplanin-deficient mice compared with cre negative controls. Scale bar = 100 μm; *n* = 4. *B*: flow cytometry analysis of podoplanin-expressing cell populations isolated from perfused lung tissue following enzymatic digestion; *n* = 7–14. *C*: flow cytometry analysis of podoplanin expression in alveolar macrophage subpopulations isolated from bronchoalveolar lavage (BAL); *n* = 7–14. One-way ANOVA performed with Tukey’s multiple comparison posttests; *P < 0.05, **P < 0.01, and ***P < 0.001. UC, unchallenged; EpCAM, epithelial cell adhesion molecule; LECs, lymphatic endothelial cells; DCs, dendritic cells; AM, alveolar macrophage.

**Fig. 6.**
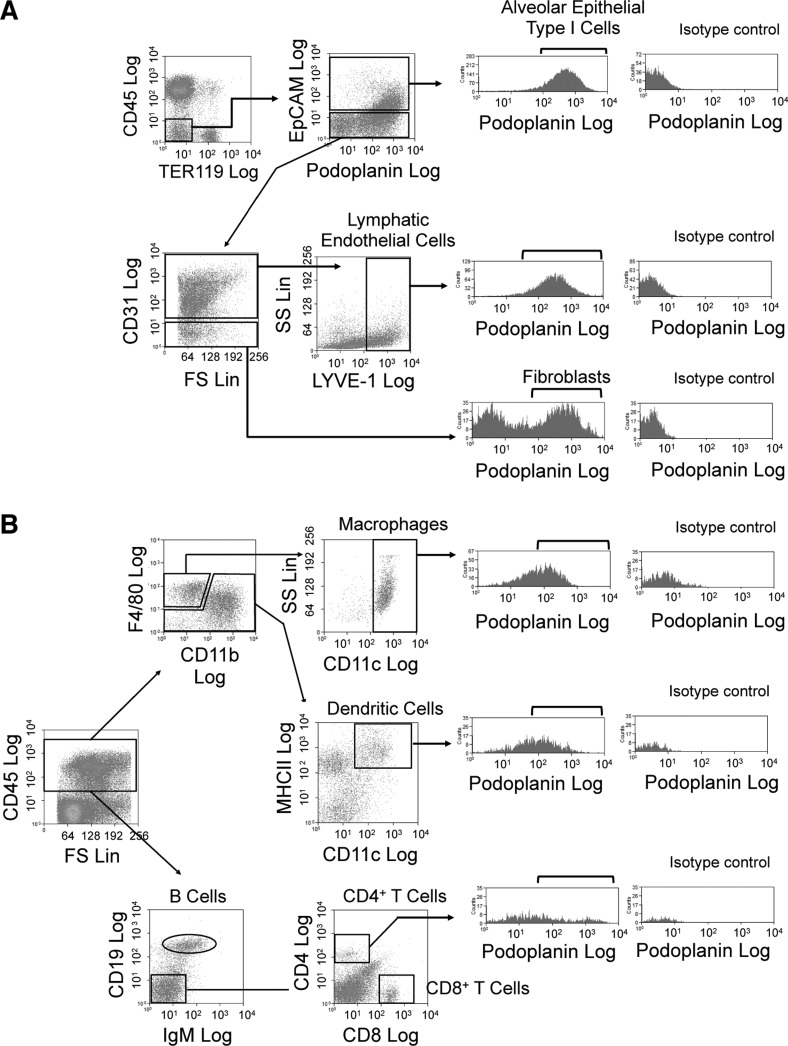
Gating strategy used in digested lung tissue. Representative histograms for the identification of podoplanin-expressing cell populations following Liberase/DNase digestion. [From Hashimoto et al. (18).] Only alive (SyTOX Red negative) cells were analyzed using a stromal (*A*) or leucocyte (*B*) antibody panel in perfused and digested mouse lungs. Podoplanin expression was analyzed by setting the gate at ≤1% by using a Syrian hamster IgG isotype as shown. Blood endothelial cells (BECs) are defined within the stromal gate as EpCAM^−^CD31^+^LYVE^−^.

To characterize these novel mouse models, we first used immunohistochemistry to evaluate podoplanin expression in unchallenged and IT-LPS-treated lungs. Deletion of podoplanin is observed only in the *Pdpn*^fl/fl^Aqp5cre^+^ lungs (Fig. 5*A*). To quantify podoplanin deletion in specific cell populations, lung tissue from unchallenged and IT-LPS-treated mice was perfused, lavage fluid isolated, and tissue enzymatically digested. Each cell population was analyzed by flow cytometry using the gating strategy shown in Fig. 6 and as previously reported (18). In digested lung tissue from unchallenged (UC) mice, we first confirmed that podoplanin in controls is expressed on AECI, lymphatic endothelial cells (LECs), fibroblasts, tissue resident macrophages, dendritic cells (DCs), and CD4^+^ T cells (Fig. 5*B*, UC white plots). Furthermore, LPS-induced lung injury significantly increases podoplanin expression on the leucocyte populations (Fig. 5*B*, 48-h white plots).

As expected, the Aqp5cre efficiently reduces podoplanin expression on AECIs (94.2 ± 0.8 to 7.5 ± 0.4%), which is maintained following LPS-induced inflammation (Fig. 5*B*, gray plots). *Pdpn*^fl/fl^Aqp5cre^+^ mice also have a significant reduction in podoplanin expression on LECs (83.9 ± 2.5 to 10.2 ± 0.5%) and tissue resident macrophages (34.1 ± 5.2 to 8.9 ± 2.8%). Podoplanin expression remains significantly reduced in these cell populations following IT-LPS. In contrast, expression of podoplanin is maintained in all lung cell populations analyzed in unchallenged *Pdpn*^fl/fl^VAV1cre^+^ lungs (Fig. 5*B*, UC black plots). However, inflammation-induced increases in podoplanin expression are inhibited in lung leucocyte populations.

The two macrophage populations present in BAL (identified in Fig. 1*A*) were also analyzed for podoplanin expression. Podoplanin is expressed at a very low level in alveolar macrophages, which does not change following IT-LPS (Fig. 5*C*, white plots). In addition, there are no significant differences in expression in both conditional podoplanin knockout strains (Fig. 5*C*, gray and black plots). This is in contrast to BAL inflammatory alveolar macrophages, which express a moderate level of podoplanin in controls (29.3% ± 3.8) and is significantly reduced only in *Pdpn*^fl/fl^VAV1cre^+^ animals (13.2% ± 2.2; Fig. 5*C*, black plots).

#### Hematopoietic-expressed podoplanin protects against LPS-induced lung injury.

**Fig. 7.**
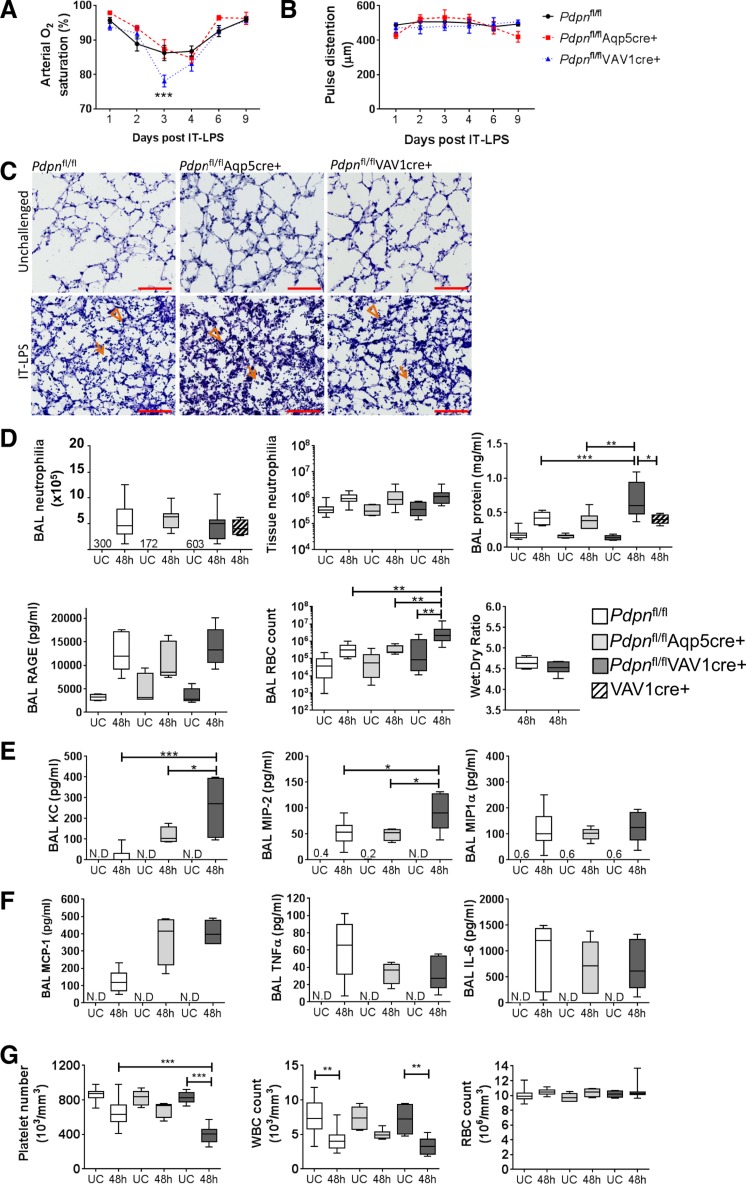
Effect of podoplanin deletion from Aqp5^+^ or VAV1^+^ cells on lung function in a mouse model of ARDS. *A*: arterial oxygen saturation analyzed by noninvasive pulse oximetry following IT-LPS in Aqp5cre- or VAV1cre-induced podoplanin-deficient mice compared with cre negative controls (*P* = 0.001). *B*: pulse distention analyzed by MouseOx Plus following IT-LPS in Aqp5cre- or VAV1cre-induced podoplanin-deficient mice compared with cre negative controls. *Pdpn*^fl/fl^ *n* = 8, *Pdpn*^fl/fl^Aqp5cre *n* = 7, *Pdpn*^fl/fl^VAV1cre *n* = 7; means ± SE. Two-way ANOVA performed with Tukey’s multiple comparisons test; ***P < 0.001. *C*: representative images of lung sections stained for hematoxylin and eosin in unchallenged and 48 h after IT-LPS treatment in Aqp5cre or VAV1cre-induced podoplanin-deficient mice compared with cre negative controls. Alveolar and interstitial neutrophils are highlighted by arrows and arrowheads, respectively. Scale bar = 100 μm; *n* = 3. *D*: neutrophil recruitment and pulmonary endothelial and epithelial damage parameters in unchallenged and IT-LPS-treated Aqp5cre- or VAV1cre-induced podoplanin-deficient mice compared with cre negative controls; *n* = 7–14. *E *and *F*: BAL cyto- and chemokine levels in unchallenged and IT-LPS-treated Aqp5cre- or VAV1cre-induced podoplanin-deficient mice compared with cre negative controls; *n *= 6. *G*: systemic platelet, white blood cell (WBC), and red blood cell counts (RBC) were analyzed in whole blood on an ABX Pentra 60 (Horbia); *n *= 6–11. One-way ANOVA performed with Tukey’s multiple comparison posttests; *P < 0.05, **P < 0.01, and ***P < 0.001. UC, unchallenged; N.D., not detected; 48 h = 48 h post-IT-LPS.

To investigate the consequence of podoplanin deletion in our conditional strains, we first analyzed $Sa_{O_{2}}$following IT-LPS. Deletion of podoplanin significantly reduced $Sa_{O_{2}}$only in *Pdpn*^fl/fl^VAV1cre^+^ compared with controls and *Pdpn*^fl/fl^Aqp5cre^+^ animals (*P* = 0.001; Fig. 7*A*), without altering blood flow (Fig. 7*B*). Immunohistochemistry analysis suggests that neutrophil recruitment following IT-LPS is unaltered in both podoplanin-deficient strains (Fig. 7*C*). This was supported by analysis of neutrophil counts in BAL and lung tissue 48 h post-IT-LPS, which confirmed that LPS-induced neutrophilia is not significantly different in either strain (Fig. 7*D*). However, in line with CLEC-2-deficient strains there is a significant increase in BAL protein in *Pdpn*^fl/fl^VAV1cre^+^ animals (1.6 fold; Fig. 7*D*). Again, this is independent of changes in wet wt-to-dry wt ratio and epithelial cell damage as assessed by expression of RAGE in BAL fluid (Fig. 7*D*). However, contrary to CLEC-2-deficient strains, the number of RBCs in BAL is significantly increased only in *Pdpn*^fl/fl^VAV1cre^+^ post-IT-LPS (Fig. 7*D*). Mice expressing VAV1cre-only did not exhibit an increase in neutrophilia or BAL protein compared with floxed-only controls 48 h post-IT-LPS, suggesting our data are specific to podoplanin deletion (Fig. 7*D*, striped plots).

Expression of the neutrophil recruitment chemokines KC and MIP-2 are both significantly increased in *Pdpn*^fl/fl^VAV1cre^+^ BAL compared with floxed-only controls and *Pdpn*^fl/fl^Aqp5cre^+^ animals, mirroring the CLEC-2-deficient strains (Fig. 7*E*). In addition, no significant difference is observed in MCP-1, TNFα, or IL-6 levels in BAL (Fig. 7*F*). The increase in BAL RBCs is mirrored by a decrease in platelet counts observed in *Pdpn*^fl/fl^VAV1cre^+^ mice following IT-LPS, whereas WBC and RBC counts are comparable to floxed-only controls (Fig. 7*G*).

## DISCUSSION

The data presented in this study *1*) confirm that platelets are present in the alveolar space during inflammation, primarily in aggregates with neutrophils; *2*) show that CLEC-2 expressed on platelets protects against excessive lung inflammation in a mouse model of ARDS; *3*) are suggestive of a nonessential, or redundant, role for the CLEC-2 ligand, podoplanin, on AECI and LECs during lung development and function during inflammation; and *4*) implicate podoplanin expression on hematopoietic-derived cells in protecting against exaggerated lung inflammation in a mouse model of ARDS.

Platelets have been implicated in regulating many processes dysregulated during ARDS, including neutrophil recruitment, macrophage-dependent inflammation, and alveolar-capillary permeability; however, the mechanisms are incompletely understood (37, 52). Previously, Ortiz-Muñoz et al. (42) used two-photon intravital microscopy to show neutrophil-platelet aggregates forming dynamically during LPS-induced inflammation, which migrate into the alveolar spaces. Here we first confirm that there is an increase in platelets present within alveoli following intratracheal LPS administration. In addition, we confirm that these platelets are primarily bound to neutrophils in BAL fluid.

Having confirmed the presence of platelet-neutrophil aggregates in the alveoli, we investigated the role of the platelet receptor CLEC-2 in regulating lung inflammation. Using two complementary strategies of CLEC-2 deletion, our data suggest that platelet CLEC-2 is protective during LPS-induced lung injury. Our results show for the first time that in a mouse model of ARDS, expression of CLEC-2 on platelets limits neutrophil extravasation into the alveolar spaces, possibly via modulation of the chemokines KC and MIP-2, maintaining lung function. In the lung, MIP-2 is primarily produced by alveolar macrophages (8), whereas KC is expressed by epithelial cells, neutrophils, and macrophages (21). Platelets also contain KC (14), so whether CLEC-2 deficiency only affects KC release from platelets or modulates expression of the inflammatory chemokines by other cell types is the focus of ongoing research.

This protective role for platelets in a mouse model of intratracheal LPS-induced lung injury contradicts recent studies which demonstrate that reduced platelet number and/or activation is protective during aerosolized LPS (16, 54), acid-induced (58), and TRALI (30, 42) mouse models. In line with these data a recent prospective study using a human model of ARDS suggests that reduced platelet activation via aspirin administration significantly reduces acute pulmonary neutrophilia (17). However, meta-analysis of clinical studies using aspirin in the management of ARDS concluded that data are insufficient to justify the use of aspirin as yet (44). Interestingly, an injurious role for platelet CLEC-2 during a mouse model of deep vein thrombosis was recently reported (46), with platelet transfusion shown to be protective in mice that were first subjected to extracorporeal circulation (34) or during mouse models of sepsis (57). Together with the data we present here that suggests platelet transfusion before IT-LPS has a detrimental effect, these data emphasize the possible model-dependent and/or receptor-dependent effects of platelets during inflammation.

The only known endogenous ligand for CLEC-2 is podoplanin, which we show is highly expressed in the lung on multiple cell types and upregulated on leucocytes during inflammation in the lung, in line with previously published data (11, 26, 43, 48, 51). We used *cre-loxP* technology to induce cell-specific deletion of podoplanin. Mice generated using Aqp5cre or VAV1cre-induced podoplanin deletion are viable and have no overt phenotype. In particular we show for the first time that podoplanin expressed on AECIs is not required for lung development. This argues against a major role of AECI-expressed podoplanin in contributing to the embryonic lethality observed in constitutive podoplanin knockout mice (50). Furthermore, we observe that use of Aqp5cre has the ability to reduce expression of the floxed gene in LECs and macrophages digested from lung tissue. Although this was not reported in the original description of the Aqp5cre mouse, this may be due to expression of Aqp5 on endothelium and leucocytes as reported in other tissues (27, 38). Furthermore, the Aqp5cre was first described on a 129S6/SvEvTac background. This newly generated strain is on a C57Bl/6 background, which may account for the differences observed.

**Fig. 8.**
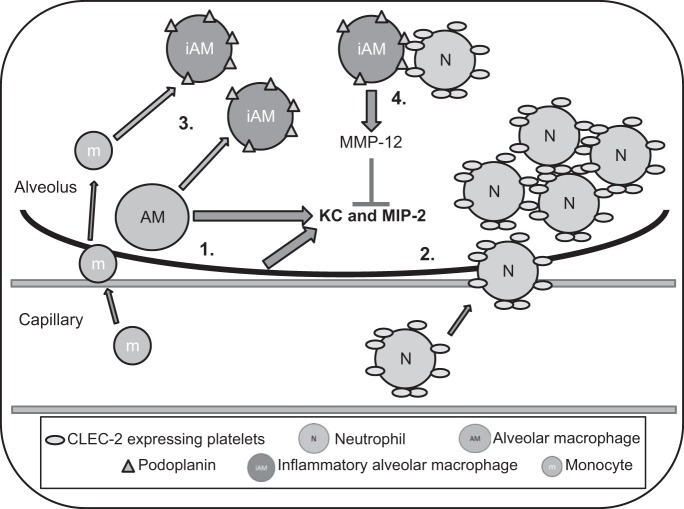
Proposed model for the mechanism of CLEC-2 podoplanin-dependent regulation of acute lung injury. During acute lung inflammation, the CXC chemokines KC and MIP-2 are produced by alveolar macrophages (AM) and epithelial cells (1). Platelets in aggregates with neutrophils (N) are recruited into the alveoli (2). At the same time, tissue resident AM and monocytes (m) from the blood contribute to the appearance of CD11b^+^ inflammatory AM (iAM) in the alveoli, which express podoplanin (3). Together in the alveolar space, CLEC-2 expressed on platelets interacts with podoplanin on the iAMs regulating MMP-12 release from iAMs (4). MMP-12 is able to cleave and inactivate the CXC chemokines contributing to the “off” signal during acute inflammation in the lung and limiting lung injury.

Using our podoplanin-deficient mouse strains, the data presented here suggest that podoplanin expressed on lung epithelial, endothelial, and tissue leucocytes does not regulate lung injury or inflammation. However, podoplanin expression on a distinct hematopoietic cell(s) is protective during LPS-induced inflammation in the lung. Furthermore, VAV1^+^ podoplanin-expressing cells limit BAL protein accumulation and alveolar hemorrhage, as well as maintain lung function, via modulation of KC and MIP-2 expression. In particular, as the only cell population with significantly lower podoplanin expression solely in *Pdpn*^fl/fl^VAV1cre^+^ mice following LPS (Fig. 5*C*), loss of podoplanin from BAL CD11b^+^CD11c^+^F4/80^+^ alveolar inflammatory macrophages may play a key role in limiting endotoxemia-induced inflammation in the lung. This subpopulation of alveolar macrophages has been reported to express high levels of matrix metalloproteinase (MMP)-12 (9), which cleaves and inactivates CXC chemokines, including KC and MIP-2, contributing to the “off” signal during acute inflammation of the lung (6). Therefore it is interesting to speculate that regulation by platelet CLEC-2 may modulate MMP-12 release/activity via direct interaction with podoplanin on these inflammatory alveolar macrophages. This possible mechanism is summarized in Fig. 8.

Differences between *Pdpn*^fl/fl^VAV1cre^+^ and CLEC-2-deficient animals were also observed. Most notably, the lack of increased BAL neutrophilia and conversely an increase in BAL RBC accumulation in the *Pdpn*^fl/fl^VAV1cre strain post-IT-LPS. There is growing evidence that podoplanin may interact with other proteins, including CCL21 and galectin 8 (4, 25). This may suggest that in *Pdpn*^fl/fl^VAV1cre^+^ animals both CLEC-2-dependent and CLEC-2-independent mechanisms are active, both of which contribute to the phenotype observed post-IT-LPS. We also cannot rule out the possibility that a stromal/fibroblast podoplanin-expressing cell contributes to the phenotype of CLEC-2-deficient mice. Future mechanistic studies are required to fully understand the role of CLEC-2 and podoplanin during lung inflammation.

Both *cre-loxP* strategies we employed to delete CLEC-2 have their limitations: *1*) use of tamoxifen-may also delete CLEC-2 in cells besides platelets, although the use of the CD11c-cre does not support this; *2*) tamoxifen is anti-inflammatory; however, parameters from our floxed-only controls of tamoxifen and nontamoxifen-treated controls are comparable; and *3*) previous reports suggest there are limitations in specificity using *cre-loxP* technology; specifically, expression of the PF4cre has recently been shown in low numbers of nonmegakaryocytic lineages (47). However, the close correlation of data generated using both strategies strongly support the validity of our findings.

A further limitation of this study is the use of only one model of ARDS, which on its own does not completely reproduce all the features of the clinical syndrome. The main feature of the IT-LPS model is alveolar neutrophilia. As a role for platelets in regulating neutrophil recruitment has already been suggested, we reasoned that IT-LPS would be the best to initially investigate the role of platelet-expressed CLEC-2. It will now be essential to confirm these findings in other mouse models of ARDS.

In conclusion, our data support the finding that CLEC-2 expressed on platelets is required to limit neutrophil recruitment, which, in turn, limits lung function decline in a mouse model of ARDS. In addition, expression of the CLEC-2 ligand podoplanin is required on hematopoietic cells to limit neutrophil chemokine expression and consequently arterial oxygen saturation decline. Therefore we *1*) demonstrate that the platelet CLEC-2-podoplanin signaling axis is a novel regulator of lung inflammation in mouse *2*), emphasize the complex role platelets play during mouse ARDS, and *3*) identify the platelet CLEC-2-podoplanin pathway as a possible novel target for therapeutic intervention in patients at risk of developing ARDS.

## GRANTS

S. Watson was supported by British Heart Foundation Grants CH/03/003 and RG/13/18/30563; S. Lax and D. Thickett were supported by Wellcome Trust Grant 091864/Z/10/Z; and Z. Borok and E. Crandall were supported by the Hastings Foundation, the Whittier Foundation, and National Heart, Lung, and Blood Institute Grants HL-126877 (to Z. Borok), HL-112638 (to Z. Borok), HL-114094 (to Z. Borok), and HL-108634 (to E. Crandall). E. Crandall is Hastings Professor and Norris Chair of Medicine. Z. Borok is Edgington Chair of Medicine.

## DISCLOSURES

No conflicts of interest, financial or otherwise, are declared by the authors.

## AUTHOR CONTRIBUTIONS

S.L., J.R., S.W., E.J.H., K.L., P.F., Z.B., E.D.C., D.T., and S.P.W. conceived and designed research; S.L., J.R., S.W., E.J.H., K.L., B.G., and R.L. performed experiments; S.L., J.R., E.J.H., K.L., B.G., and R.L. analyzed data; S.L., J.R., S.W., E.J.H., K.L., and B.G. interpreted results of experiments; S.L. prepared figures; S.L. drafted manuscript; S.L., J.R., S.W., E.J.H., K.L., B.G., R.L., P.F., Z.B., E.D.C., D.T., and S.P.W. edited and revised manuscript; S.L., J.R., S.W., E.J.H., K.L., B.G., R.L., P.F., Z.B., E.D.C., D.T., and S.P.W. approved final version of manuscript.
